# Design and Analysis of Intelligent Robot Based on Internet of Things Technology

**DOI:** 10.1155/2022/7304180

**Published:** 2022-05-12

**Authors:** Yunfeng Yao, Suling Li

**Affiliations:** ^1^College of Mechanical and Electrical Engineering, Jiaxing Nanhu University, Jiaxing, Zhejiang 314001, China; ^2^Nanchang Institute of Technology, Nanchang, Jiangxi 330044, China

## Abstract

This research uses Auto-ID Labs radio frequency identification system to realize the information dissemination from the destination node to the nodes in its neighborhood. The purpose is to forward messages and explore typical applications. Realize the intelligent analysis and management of IoT devices and data. Design a set of edge video CDN system, in the G1 data set *A* = 9, *p* = 9, ℤp = 9, *l*ℤp = 8, AES = 5, ES = 9. Distribute some hot content to public wireless hotspots closer to users in advance, *A* = 9, *p* = 7, ℤp = 9, *l*ℤp = 9, AES = 9, ES = 8. At present, a large amount of research is mainly to deploy an edge node between the end node of the Internet of Things and the cloud computing center to provide high-quality services. By learning a stable dynamic system from human teaching to ensure the robustness of the controller to spatial disturbances. FPP-SCA plan FPP-SCA = 1.99, FPP-SCA = 1.86, FPP-SCA = 1.03, FPP-SCA = 1.18, FPP-SCA = 1.01, FPP-SCA = 1.46, FPP-SCA = 1.61.The more robots work in an unstructured environment, with different scenarios and tasks, the comparison shows that the FPP-SCA scheme is the optimal model F-S0 = 2.52, F-S5 = 2.38, F-S10 = 2.5, F- S15 = 2.09, F-S20 = 2.54, F-S25 = 2.8, F-S30 = 2.98.

## 1. Introduction

The Internet of Things (IoT) connects various sensors to the Internet to realize the intelligent analysis and management of Internet of Things equipment and data. Information dissemination of nodes in the neighborhood. Its concept was formally confirmed in the report of the 2005 International Telecommunication Union World Summit on the Information Society. Point out that the node that will receive this message performs intelligent analysis and manages the broadcast data message. The node receiving this message judges whether it is between the destination node and the source node [1–3]. In the first stage, the message is forwarded, mainly for the purpose of exploring typical applications. At this stage, the data is sensed and obtained by the information terminal node of the Internet of Things, and is transmitted to an independent and centralized data center for processing through the energy multi-path protocol multi-hop mode. However, due to the limitation of node energy and communication energy consumption technology at this stage, a large number of node energy and communication energy consumption applications cannot be implemented well. For example, the clustered hierarchical deployment algorithm adopted by the wireless sensor network cannot undertake the real-time transmission of large-capacity data such as video data. Only by controlling the routing hop count to avoid the loss of transmission data [4–6]. The information is transmitted to the cluster head in the communication time slot, and the cluster head collects a small amount of data such as humidity. And during large-scale deployment, the difficulty of application deployment, development, and debugging has increased significantly. In terms of the number of nodes, the total installed capacity of the energy multi-path routing protocol global Internet of Things equipment is about 9.1 billion, and it will reach 28.1 billion later. With the emergence of a new computing model of transmission path control edge computing and the development of hardware, the development of the Internet of Things has entered a new stage in order to solve the growing problems of Internet of Things data and meet the needs of low-latency services. In the stage of clustering edge node algorithm with better real-time performance, the edge node protocol is introduced into the optimization step system. The IoT data computing tasks originally deployed in the cloud computing center are migrated to hierarchical energy multi-path sensing nodes and edge nodes. IoT data is processed at the edge of the network, and only a small amount of data needs to be transmitted to the cloud computing center for final analysis and storage. The operation of the task design process of the online scheduling algorithm of the robot mobile node can be divided into: grasping, palletizing, polishing, polishing, welding, and dispensing according to the task type. However, at the execution level of the robot, an important problem is the task conflict between mobile nodes. The most basic and common operation skills can be divided into point-to-point motion (corresponding task scenarios include palletizing, grabbing, and placing, etc.), which is the intelligent analysis of multi-threaded tasks in the scene when access conflicts occur in the online scheduling algorithm. Select and perform algorithmic scheduling on nodes. Trajectory tracking movement (corresponding task scenarios include welding, dispensing, etc.), and game theory is commonly used to solve the problem of trajectory tracking movement conflicts. Smooth motion with mixed force and position (corresponding task scenes include polishing, sanding, etc.) [7–13]. The study of robot point-to-point motion learning is based on visually guided hand-eye coordinated motion. Point motion algorithm status For intelligent multi-purpose robots, the online scheduling algorithm of mobile nodes can be realized by executing a set of pre-programmed actions. However, it is not enough to rely on the execution of a set of pre-programmed actions or behaviors of the point motion algorithm, once disturbances (such as deviations of position and displacement) occur. The robot's operation task may fail due to calculation errors in positioning. Robot demonstration teaching involves learning the corresponding task scenes, including visually guided object grabbing and assembly. Teaching gives the robot basic skills in teaching (dragging teaching, remote operation teaching, and observation (video, image teaching information extraction) teaching, etc.). The ability to learn how to perform teaching tasks in the online scheduling algorithm of mobile nodes in the game theory method. The control strategy completes formation maintenance [13–15]. The biggest difference between this method and the traditional method is that it avoids the situation of large transmission delay, and the clustering algorithm has better real-time performance. The dynamic system (DS) modeling the robot motion is to allow the robot to understand and comprehend a certain operation skill, and still have a certain generalization ability to perform motion coding under new conditions, not just simple repetition. The dynamic energy multi-path routing protocol system is used to encode the robot motion characteristics. It allows the discarded message to adapt to changes in the dynamic environment, and reproduce the movement taught in the optimization step, following the path defined by the pre-forwarding node's measurement standard. Many scholars use dynamic systems to control the formation of nodes through the optimal data transmission path of the machine. Introduce node energy and communication energy consumption as hand-eye coordinated movement based on visual guidance. Choosing the number of hops avoids the situation where the task scene has a large transmission delay for visually guided objects to grab and transmit. Point-to-point motion clustering algorithm with better real-time performance, using trajectory tracking motion formation control strategy to complete formation maintenance. The compliant motion of force and position mixing is based on clustering, and the hierarchical algorithm divides the signal network into several clusters based on visual guidance of hand-eye coordinated motion according to a certain rule. Learning method and intelligent control technology The cluster transmits information by the cluster head and the communication time slot within the cluster.

## 2. Dynamic Architecture of IoT Technology

### 2.1. Architecture of Dynamic Secure Collaborative Computing Method under VECA

**Figure 1:**
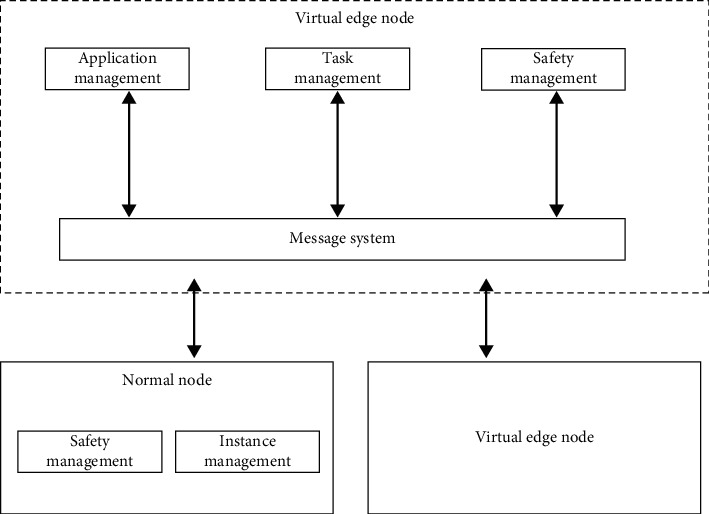
Architecture of dynamic secure collaborative computing method under VECA.

The architecture of the dynamic secure computing method includes virtual edge nodes composed of application management, task management, security management, and message systems. Common nodes composed of security management and instance management, virtual edge nodes and common nodes realize interconnection and intercommunication. The dynamic security calculation method transmits information to the virtual edge node in the corresponding security management and instance management communication time slots. After collecting and fusing information between ordinary nodes, it is transmitted to task management. The virtual edge node has a heavier load than other nodes. After a round of data transmission is completed, a new round of secure calculation method architecture will be performed to balance energy consumption. As shown in Figure 1.

### 2.2. General Process Based on CNN Training and Testing

**Figure 2:**
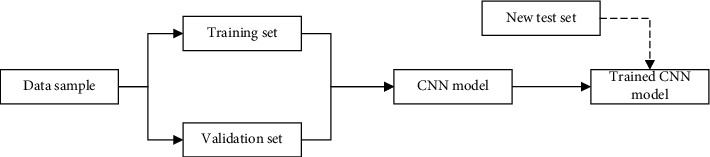
CNN training test.

The CNN training and testing method is to realize the collection of data samples through the online scheduling algorithm of mobile nodes. Resolve task conflicts between mobile nodes on the training set and data validation set encountered during the design process. Access conflicts caused by task conflicts between mobile nodes are imported into the CNN model for training and new test set collection, the results are classified and verified, and the correctness and error rates of the results are verified. The method often involved in resolving conflicts is the line scheduling algorithm. It mainly includes the application of analyzing wireless sensor networks to model the interaction between players with conflicting interests. To compete for limited network resources such as energy and bandwidth. as shown in Figure 2.

### 2.3. Joint Transmission of Information and Power

The battery capacity of wireless information and power combined transmission sensor equipment is limited. In order to save costs and facilitate the distribution of its battery capacity, the battery capacity is usually relatively small, and it is inconvenient to replace the battery, which may be dangerous, costly, and sometimes not feasible. In the natural environment, energy exists in many forms, is widely distributed, and has infinite supply. It can be obtained, developed and utilized in large quantities without any extra effort. However, the implementation of energy harvesting technology is not simple. In order to effectively convert energy and effectively use energy, many factors need to be considered. Since each dimension is separated in the modeling process, it is difficult to generalize a learned dynamic system to other different shapes of motion. The stability estimation of dynamic system (SEDS) method is to ensure the robustness of the controller to spatial disturbances by learning a stable dynamic system from human teaching. SEDS uses Gaussian mixture model to model the motion, and ensures the global asymptotic stability of the dynamic system by introducing stability constraints derived from the Lyapunov function. The SEDS method provides a theoretical framework to ensure the global asymptotic stability of the learning dynamic system. This method has certain reference significance for solving the robustness problem of the nonlinear dynamic system.

## 3. Application of IoT Technology in Smart Robots

### 3.1. FPP-SCA [16–19]

Information dissemination from the destination node to the nodes in its neighborhood

(1) $$\begin{array}{c} X_{j}=X_{i}+\text{visual}*\text{rand}\left(\;\right)\text{.} \end{array}$$

CDN system

(2) $$\begin{array}{c} X_{i}^{+}=X_{i}+\text{rand}\left(\;\right)*\text{step}*\frac{X_{j}-X_{i}}{\left\|X_{j}-X_{i}\right\|},\\ X_{i}^{+}=X_{i}+\text{rand}\left(\;\right)*\text{step}*\frac{X_{m}-X_{i}}{\left\|X_{m}-X_{i}\right\|}. \end{array}$$

IoT devices

(3) $$\begin{array}{c} \text{visual}=V_{\max}-\frac{V_{\max}-V_{\min}}{i_{\max}}*i,\\ \text{step}=S_{\max}-\frac{S_{\max}-S_{\min}}{i_{\max}}*i. \end{array}$$

Intelligent analysis of IoT data

(4) $$\begin{array}{c} F={\left[\frac{1}{N}\sum_{i}^{N}\sum_{j}^{K}{\left(O_{ij}-y_{ij}\right)}^{2}\right]}^{-1}. \end{array}$$

The Internet of Things in the Cloud Computing Center

(5) $$\begin{array}{c} \beta =H_{1}\left(c_{1},c_{2},c_{3},c_{4},c_{5},ke{\left(h_{0},g_{3}\right)}^{t}\right),\\ CT=\left\{c_{1},c_{2},c_{3},c_{4},c_{5},\beta ,S,{\text{SEnc}}_{\text{key}}\left(M\right)\right\},\\ \text{rct}=\text{Enc}\left(\mu ,S\prime ,r,{\text{fun}}_{r}\right). \end{array}$$

### 3.2. SNRS [20–23]

(6) $$\begin{array}{c} u_{i,r,0}=g_{2}^{r_{i},r,1}R^{-1},\\ RK_{i,S⟶S^{\prime },r}=\left\{u_{i,r,-1},u_{i,r,0},u_{i,r,1},\ldots ,u_{i,r,n},\text{rct},S\right\}. \end{array}$$

Robot execution layer

(7) $$\begin{array}{c} ID_{j}\in S\left(j=1,2,\ldots ,n\right),\\ c=\frac{e\left(\prod_{j=1}^{n}u_{i,r,j},c_{2}\right)}{c_{3}^{u_{i,r,-1}}c_{4}^{f\left(u_{i,r,-1}\right)}e\left(c_{1},u_{i,r,0}\right)}. \end{array}$$

Hierarchical energy multipath perception

(8) $$\begin{array}{c} CT^{\prime }=\left\{c_{1},c_{2},c_{3},c_{4},c_{5},\beta ,c_{6},\text{rct},S^{\prime },{\text{SEnc}}_{\text{key}}\left(M\right)\right\}. \end{array}$$

Intelligent sorting

(9) $$\begin{array}{c} e{\left(h_{0},g_{3}\right)}^{t}=\frac{e\left(\prod_{j=1}^{n}u_{i,r,j},c_{2}\right)}{c_{3}^{u_{i,r,-1}}c_{4}^{f\left(u_{i,r,-1}\right)}e\left(c_{1},u_{i,r,0}\right)},\\ \overset{\wedge }{s}=H_{1}\left(c_{1},c_{2},c_{3},c_{4},e{\left(h_{0},g_{3}\right)}^{t}\right),\\ ke{\left(h_{0},g_{3}\right)}^{t}=\frac{c_{5}}{e{\left(h_{0},g_{3}\right)}^{\overset{\wedge }{s}}}. \end{array}$$

Intelligent management of IoT data

(10) $$\begin{array}{c} k=\frac{ke{\left(h_{0},g_{3}\right)}^{t}}{e{\left(h_{0},g_{3}\right)}^{t}},\\ e{\left(h_{0},g_{3}\right)}^{t_{d-1}}=\frac{c_{6}^{d-1}}{e\left(c_{1}^{d-1},R_{d}^{-1}\right)}. \end{array}$$

### 3.3. SDR [24, 25]

(11) $$\begin{array}{c} P_{\text{avg}}=\sum_{i=1}^{N}\frac{C_{i}V{\left(\alpha \right)}^{2}AF\left(\alpha \right)}{2}. \end{array}$$

Robot demonstration teaches learning

(12) $$\begin{array}{c} M=\frac{B-\min\left(B\right)}{\max\left(B\right)-\min\left(B\right)},\\ \text{precision}\left(i\right)=\frac{{\text{TP}}_{i}}{\left({\text{TP}}_{i}+{\text{FP}}_{i}\right)}. \end{array}$$

Robot trajectory tracking

(13) $$\begin{array}{c} \text{recall}\left(i\right)=\frac{{\text{TP}}_{i}}{\left({\text{TP}}_{i}+{\text{FN}}_{i}\right)}. \end{array}$$

Edge computing

(14) F1−Scorei=2×Pri×ReiPri×Rei,ΦB=μNIScos  θ.

Robot teaches from

(15) $$\begin{array}{c} \log\;f_{0}\left(e^{y}\right),\\ \log\;f_{0}\left(e^{y}\right)\leq 0,\;i=1,\ldots ,m,\\ \log\;h_{1}\left(e^{y}\right)\leq 0,\;i=1,\ldots ,M,\\ C=B\times \log_{2}\;\det\left(I_{N_{R}}+\frac{{\text{E}}_{s}HH^{H}}{N_{0}}\right). \end{array}$$

## 4. Simulation Experiment

### 4.1. Edge Computing

**Figure 3:**
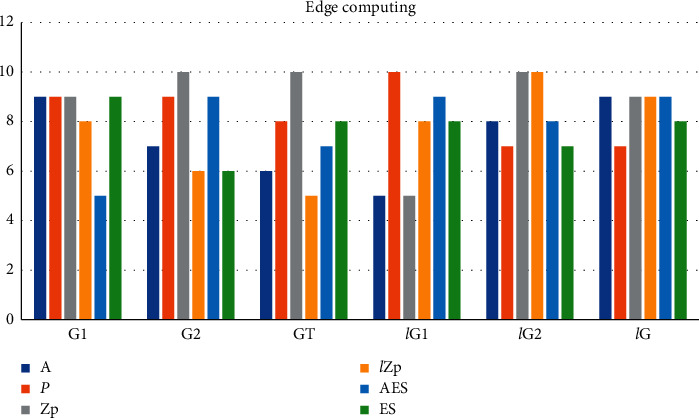
Edge computing.

The research work of edge computing revolves around data downlink services. A set of edge video CDN system is designed, in the G1 data set *A* = 9, *p* = 9, ℤp = 9, *l*ℤp = 8, AES = 5, ES = 9. Distribute some hot content to public wireless hotspots closer to users in advance, *A* = 9, *p* = 7, ℤp = 9, *l*ℤp = 9, AES = 9, ES = 8. At present, a large amount of research is mainly to deploy an edge node between the end node of the Internet of Things and the cloud computing center to provide high-quality services. As shown in Table 1 and Figure 3.

### 4.2. TSP Algorithm

**Figure 4:**
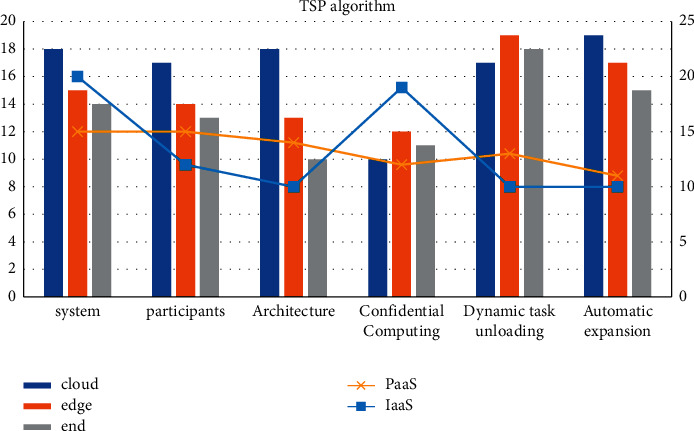
TSP algorithm.

Regarding the multi-route IoT nested non-cooperative problem, even if it is not a large-scale network individual, it may be selfish. The number of variables is also very large, and the solution is in the best interest. It is very difficult to directly deviate from the established solution. The conversion to the energy-constrained TSP algorithm is to convert the refusal forwarding mTSP into the standard freight forwarding dilemma TSP, which can solve the pure strategy set Si and the revenue function µi. The algorithm designed for TSP can solve it by deviating from the Nash equilibrium. The basic parameter variables are shown in Table 2 and Figure 4.

### 4.3. Data Values of Different Scenarios

**Figure 5:**
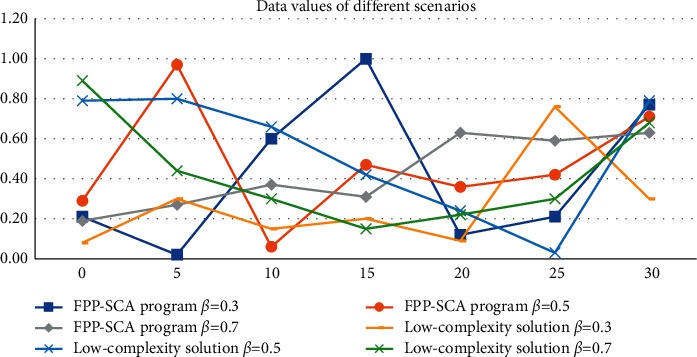
Data values of different scenarios.

Energy model In order to effectively complete the data transmission and charging tasks of the sensor node, the mobile node needs to reach the sensor node for energy replenishment before the sensor node does not have enough power to continue working. In the FPP-SCA scheme, *β* = 0.3 SNRs is 0.21, *β* = 0.5 SNRs is 0.29, *β* = 0.7 SNRs is 0.19, *β* = 0.3 SNRs is 0.08, *β* = 0.5 SNRs is 0.79, *β* = 0.7 SNRs is 0.89. When a sensor node receives a beacon signal from a mobile node in the distance-based energy loss model, according to the strength of the beacon signal, the distance required for the sensor node to send data to the mobile node can be estimated. When *β* = 0.3, the SNRs is 0.77, When *β* = 0.5, SNRs is 0.71, when *β* = 0.7, SNRs is 0.63, when *β* = 0.3, SNRs is 0.3, when *β* = 0.5, SNRs is 0.79, and when *β* = 0.7, SNRs is 0.68. As shown in Table 3 and Figure 5.

**Figure 6:**
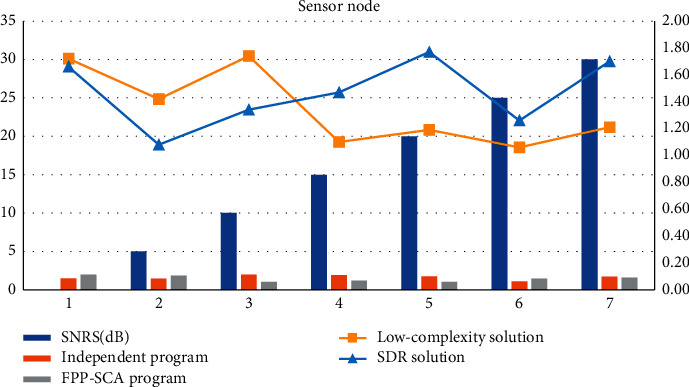
Sensor node.

The location of sensor nodes can be known in advance. Independent scheme, FPP-SCA scheme, low-complexity scheme, SDR scheme. These nodes are used as relays to collect the information monitored by the static nodes and charge these sensor nodes. In the independent scheme, *S* = 0, ID = 1.51, *S* = 5, ID = 1.47, *S* = 10, ID = 1.98, *S* = 15, ID = 1.92, *S* = 20, ID = 1.77, *S* = 25, ID = 1.09. By learning a stable dynamic system from human teaching to ensure the robustness of the controller to spatial disturbances. FPP-SCA scheme FPP-SCA = 1.99, FPP-SCA = 1.86, FPP-SCA = 1.03, FPP-SCA = 1.18, FPP-SCA = 1.01, FPP-SCA = 1.46, FPP-SCA = 1.61. As shown in Table 4 and Figure 6.

### 4.4. Model Comparison

**Figure 7:**
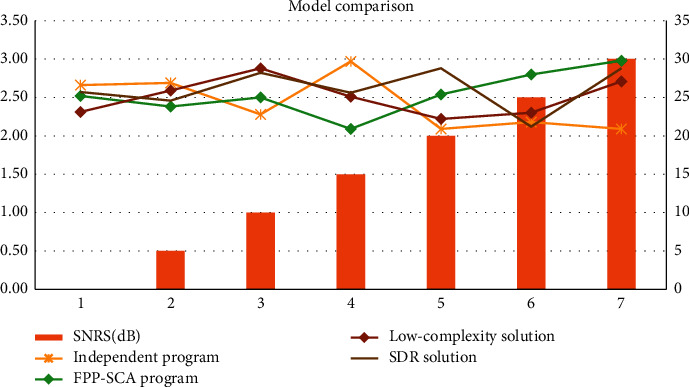
Model comparison.

Comparing the independent scheme, FPP-SCA scheme, low complexity scheme, and SDR, it is found that the global asymptotic stability can ensure that the system responds appropriately and quickly to the disturbances that the robot may encounter during the movement. More and more robots work in unstructured environments, with different scenarios and tasks. The comparison shows that the FPP-SCA scheme is the optimal model F-S0 = 2.52, F-S5 = 2.38, F-S10 = 2.5, F-S15 = 2.09, F-S20 = 2.54, F-S25 = 2.8, F-S30 = 2.98. As shown in Table 5 and Figure 7.

**Figure 8:**
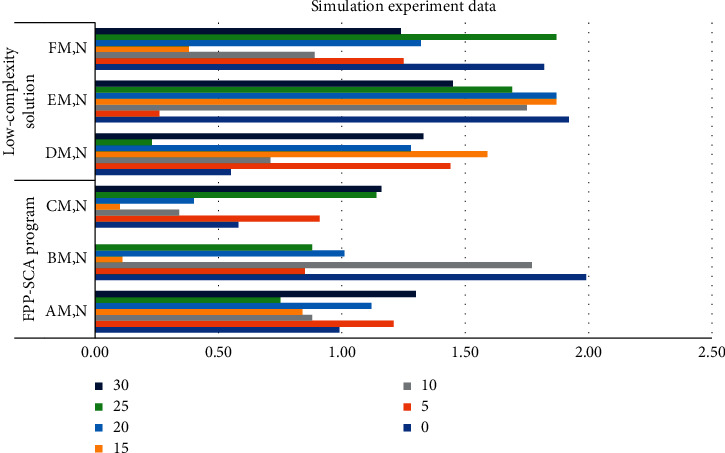
Simulated experimental data.

Robots need to master new skills frequently and quickly. Therefore, the learning speed of the algorithm is also an important evaluation index for the study of teaching learning algorithms. Fast learning algorithms can make sports learning more efficient. In the FPP-SCA scheme model, AM, *N* = 0.99, BM, *N* = 1.99, CM, *N* = 0.58, DM, *N* = 0.55, EM, *N* = 1.92, FM, *N* = 1.82. The FPP-SCA project model uses dynamic motion primitives to improve the accuracy of DMP modeling. An autonomous dynamic system model of human motion is established, and stability is achieved through a combination of linear and nonlinear dynamic systems. The dimensions of low complexity algorithms are all separated, AM, *N* = 1.3, BM, *N* = 0, CM, *N* = 1.16, DM, *N* = 1.33, EM, *N* = 1.45, FM, *N* = 1.24. As shown in Table 6 and Figure 8.

## 5. Conclusion

This research uses Auto-ID Labs radio frequency identification system to realize the information dissemination from the destination node to the nodes in its neighborhood. The purpose is to forward messages and explore typical applications. Realize the intelligent analysis and management of IoT devices and data.

Design a set of edge video CDN system, in the G1 data set *A* = 9, *p* = 9, ℤp = 9, *l*ℤp = 8, AES = 5, ES = 9. Distribute some hot content to public wireless hotspots closer to users in advance, *A* = 9, *p* = 7, ℤp = 9, *l*ℤp = 9, AES = 9, ES = 8. At present, a large amount of research is mainly to deploy an edge node between the end node of the Internet of Things and the cloud computing center to provide high-quality services.

## Figures and Tables

**Table 1:** Edge computing.

	A	p	ℤp	*l*ℤp	AES	ES

*𝔾*1	9	9	9	8	5	9
*𝔾*2	7	9	10	6	9	6
*𝔾*T	6	8	10	5	7	8
*l𝔾*1	5	10	5	8	9	8
*l𝔾*2	8	7	10	10	8	7
*l𝔾*	9	7	9	9	9	8

**Table 2:** TSP algorithm.

	System	Participants	Architecture	Confidential computing	Dynamic task unloading	Automatic expansion

Cloud	18	17	18	10	17	19
Edge	15	14	13	12	19	17
End	14	13	10	11	18	15
PaaS	15	15	14	12	13	11
IaaS	20	12	10	19	10	10

**Table 3:** Data values of different schemes.

	FPP-SCA program			Low-complexity solution		
SNRS(dB)	*β* = 0.3	*β* = 0.5	*β* = 0.7	*β* = 0.3	*β* = 0.5	*β* = 0.7

0	0.21	0.29	0.19	0.08	0.79	0.89
5	0.02	0.97	0.27	0.30	0.80	0.44
10	0.60	0.06	0.37	0.15	0.66	0.30
15	1.00	0.47	0.31	0.20	0.42	0.15
20	0.12	0.36	0.63	0.09	0.24	0.22
25	0.21	0.42	0.59	0.76	0.03	0.30
30	0.77	0.71	0.63	0.30	0.79	0.68

**Table 4:** Sensor nodes.

SNRS(dB)	Independent program	FPP-SCA program	Low-complexity solution	SDR solution

0	1.51	1.99	1.72	1.66
5	1.47	1.86	1.42	1.08
10	1.98	1.03	1.74	1.34
15	1.92	1.18	1.10	1.47
20	1.77	1.01	1.19	1.77
25	1.09	1.46	1.06	1.26
30	1.72	1.61	1.21	1.70

**Table 5:** Model comparison.

SNRS(dB)	Independent program	FPP-SCA program	Low-complexity solution	SDR solution

0	2.66	2.52	2.31	2.57
5	2.69	2.38	2.59	2.46
10	2.28	2.50	2.88	2.82
15	2.97	2.09	2.51	2.56
20	2.09	2.54	2.22	2.88
25	2.18	2.80	2.30	2.12
30	2.09	2.98	2.71	2.88

**Table 6:** Simulation experiment data.

	FPP-SCA program			Low-complexity solution		
SNRS(dB)	A^M,N^	B^M,N^	C^M,N^	D^M,N^	E^M,N^	F^M,N^

0	0.99	1.99	0.58	0.55	1.92	1.82
5	1.21	0.85	0.91	1.44	0.26	1.25
10	0.88	1.77	0.34	0.71	1.75	0.89
15	0.84	0.11	0.10	1.59	1.87	0.38
20	1.12	1.01	0.40	1.28	1.87	1.32
25	0.75	0.88	1.14	0.23	1.69	1.87
30	1.30	0.00	1.16	1.33	1.45	1.24

## Data Availability

The experimental data used to support the findings of this study are available from the corresponding author upon request.
